# The Effect of Quercetin on the Osteogenesic Differentiation and Angiogenic Factor Expression of Bone Marrow-Derived Mesenchymal Stem Cells

**DOI:** 10.1371/journal.pone.0129605

**Published:** 2015-06-08

**Authors:** Yuning Zhou, Yuqiong Wu, Xinquan Jiang, Xiuli Zhang, Lunguo Xia, Kaili Lin, Yuanjin Xu

**Affiliations:** 1 Department of Oral Surgery, Shanghai Ninth People's Hospital, Shanghai Jiao Tong University School of Medicine, Shanghai Key Laboratory of Stomatology and Shanghai Research Institute of Stomatology, Shanghai, China; 2 Department of Prosthodontics, Shanghai Ninth People's Hospital, Shanghai Jiao Tong University School of Medicine, Shanghai, China; 3 Oral Bioengineering Lab, Shanghai Research Institute of Stomatology, Shanghai Ninth People's Hospital, Shanghai Jiao Tong University School of Medicine, Shanghai, China; 4 Center of Craniofacial Orthodontics, Department of Oral and Cranio-maxillofacial Science, Shanghai Ninth People's Hospital, Shanghai Jiao Tong University School of Medicine, Shanghai, China; 5 Biomaterials & Tissue Engineering Research Center, Shanghai Institute of Ceramics, Chinese Academy of Sciences, Shanghai, China; 6 Laboratory of Oral Biomedical Science and Translational Medicine, School of Stomatology, Tongji University, Shanghai, China; Instituto Butantan, BRAZIL

## Abstract

Bone marrow-derived mesenchymal stem cells (BMSCs) are widely used in regenerative medicine in light of their ability to differentiate along the chondrogenic and osteogenic lineages. As a type of traditional Chinese medicine, quercetin has been preliminarily reported to promote osteogenic differentiation in osteoblasts. In the present study, the effects of quercetin on the proliferation, viability, cellular morphology, osteogenic differentiation and angiogenic factor secretion of rat BMSCs (rBMSCs) were examined by MTT assay, fluorescence activated cell sorter (FACS) analysis, real-time quantitative PCR (RT-PCR) analysis, alkaline phosphatase (ALP) activity and calcium deposition assays, and Enzyme-linked immunosorbent assay (ELISA). Moreover, whether mitogen-activated protein kinase (MAPK) signaling pathways were involved in these processes was also explored. The results showed that quercetin significantly enhanced the cell proliferation, osteogenic differentiation and angiogenic factor secretion of rBMSCs in a dose-dependent manner, with a concentration of 2 μM achieving the greatest stimulatory effect. Moreover, the activation of the extracellular signal-regulated protein kinases (ERK) and p38 pathways was observed in quercetin-treated rBMSCs. Furthermore, these induction effects could be repressed by either the ERK inhibitor PD98059 or the p38 inhibitor SB202190, respectively. These data indicated that quercetin could promote the proliferation, osteogenic differentiation and angiogenic factor secretion of rBMSCs in vitro, partially through the ERK and p38 signaling pathways.

## Introduction

Mesenchymal stem cells (MSCs) are important members of the stem cells family [1]. MSCs are ideal stem cells for tissue regeneration due to their excellent capacities for proliferation and differentiation. After being induced in vitro or in vivo, MSCs can be differentiated into several types of tussues such as fat, muscle, bone, cartilage, tendon, ligament, nerve and liver tissue [2, 3]. As a type of MSCs, bone marrow-derived mesenchymal stem cells (BMSCs) are widely used in studies of bone regeneration due to their properties of multipotency and active proliferation [4, 5]. More importantly, using appropriate methods, BMSCs can also be induced to secrete angiogenic factors, and these factors can then effect on resident vascular endothelial cells to promote blood vessel formation in vivo. The regeneration of bone defects specifically consists of a series of complex processes that are regulated by a variety of cytokines and biological signals. In a bone defect, angiogenesis occurs earlier than osteogenesis; since bone regeneration cannot successfully occur without a blood supply reaching the bone defect, angiogenesis is important for bone regeneration in vivo [6–10]. It is reported that angiogenesis is the foundational step in bone regeneration, specifically in calvarial and limb bone defects [11–13]. It is shown that angiogenesis occurs before osteogenesis in the healing of bone defects. In particular, angiogenesis provides the blood supply, which benefits the subsequent progression of osteogenesis [11]. A previous study in a rabbit calvarial defect model further revealed that there was intimate spatial and temporal correlation between newly formed blood vessels and extra skeletal bone formation [12]. In these two processes, angiogenesis occurs before osteogenesis in bone healing; then, both angiogenesis and osteogenesis participate in the bone regeneration and promote the effects of each other [13]. As shown in previous studies, many approaches have been applied to stimulate angiogenesis in the process of bone healing, including the combination with endothelial cells, the application of vascular growth factors, vascular remodeling by microsurgery and gene transfection techniques [14–17]. Therefore, the most ideal induction method would simultaneously promote the osteogenic differentiation of BMSCs and enhance the expression of angiogenic factors.

Interestingly, Chinese medicine, which could promote the secretion of osteogenic factors such as collagen type 1 (COL1), bone morphogenetic protein 2 (BMP-2), osteocalcin (OCN) and osteopontin (OPN), and the gene expression of BMP-4, runt-related transcription factor 2 (Runx2), OCN and OPN, has been increasingly applied in osteogenic induction research [18–20]. Quercetin is a type of bioflavonoid; bioflavonoids comprise numerous natural compounds, such as catechin, quercetin and rutin, among others. Quercetin is abundant in Ginkgo biloba extracts and in the flowers and leaves of many plants and fruits [21]. As a traditional Chinese medicine, quercetin has been extensively studied due to its potential pharmacological properties and beneficial health effects [22, 23], and it was firstly used as a Chinese medicine for the treatment of scurvy and heart disease [24, 25]. Recent studies have reported that quercetin could enhance the osteogenic differentiation of adipose-derived stem cells (ASCs) and osteoblastic MC3T3-E1 cells and inhibit osteoclastogenesis in RAW 264.7 cells [26–28]. Moreover, it has been reported that quercetin could stimulate Osterix (Osx), Runx2, BMP-2, OPN, OCN, COL1 and ALP gene expression in ASCs [26, 27], and increase bone sialoprotein (BSP) and OCN gene expression in osteoblastic MC3T3-E1 cells [28]. Based on these previous studies, the present study intended to evaluate the osteogenic differentiation of BMSCs as well as the expression of angiogenic factors by these cells in response to treatment with the traditional Chinese medicine quercetin.

It is well known that mitogen-activated protein kinase (MAPK) signaling pathways are involved in cell survival, proliferation and differentiation [29, 30], and comprise the extracellular signal-regulated protein kinases (ERK), p38 and c-Jun N-terminal kinase (JNK) signaling pathways. A previous study has demonstrated that these three signaling pathways are specifically involved in the osteogenic differentiation of stem cells [31]. It has also been shown that the ERK signaling pathway can stimulate the osteogenic differentiation of osteoblasts [32] and that the JNK and p38 signaling pathways can promote the synthesis of extracellular matrix and calcium salt deposits. Previous studies have additionally shown that the ERK signaling pathway is involved in the drug effects of quercetin on MG-63 human osteoblasts and human ASCs [26, 33]. However, it remains to be evaluated whether ERK activation is achieved through the effects of quercetin, which include cell proliferation, osteogenic differentiation and angiogenic factor expression, on BMSCs. Moreover, it should be confirmed whether the other two signaling pathways (p38 and JNK) are also involved in these processes.

In the present study, our hypothesis is that quercetin can induce the osteogenic differentiation of BMSCs as well as the expression of angiogenic factors in a dose-dependent manner and that these effects may be related to MAPK signaling pathways. To assess our hypothesis, quercetin was applied to rBMSCs at different concentrations, and the effects of quercetin on the proliferation and osteogenic differentiation of rBMSCs as well as the expression of angiogenic factors, were systematically investigated. Moreover, the mechanisms involved, including the ERK, JNK and p38 MAPK signaling pathways, were explored.

## Materials and Methods

### Ethics statement

All animal experiments were approved by the Ethical Committees for Animal Research of Shanghai Ninth People’s Hospital Affiliated to Shanghai Jiao Tong University, School of Medicine (Protocol Number: HKDL[2013]50).

### Isolation and culture of rBMSCs

In the present study, rBMSCs were isolated from 4-week-old male SD rats weighing 50 ± 5 g. A total of 20 rats were used for the in vitro research in the present study. The rats were sacrificed using an overdose of chloral hydrate, and then both ends of the femurs were cut off at the metaphyses. The marrow was flushed out with 10 mL Dulbecco’s modified Eagle’s medium (DMEM; Gibco, USA) supplemented with 10% fetal bovine serum (FBS; Gibco, USA) and antibiotics (penicillin 100 U/mL, streptomycin 100 U/mL) using a 22-gauge needle [34]. The primary cells were then cultured in an incubator at 37°C and 5% CO_2_ for 4 days and the medium was renewed every 2 days. When 90% confluence was reached, the rBMSCs were washed with phosphate-buffered saline (PBS) and passaged using 0.25% trypsin/ethylenediaminetetraacetic acid (trypsin/EDTA). In the present study, the cells from passages 1 to 3 were used for subsequent experiments.

### Quercetin treatment and MTT assay

Quercetin (C_15_H_10_O_7_, CAS No.: 117-39-5, Sigma, USA) was dissolved in dimethylsulfoxide (DMSO; Sigma, USA) to obtain a 20 mM stock solution, which was then diluted in the medium to desired concentrations. The MTT assay was used to test the effect of quercetin on cell proliferation. Briefly, the cells were seeded in 96-well plates at a density of 5×10^3^ cells/well and cultured in DMEM (Gibco, USA) supplemented with 10% FBS (Gibco, USA) and antibiotics (penicillin 100 U/mL, streptomycin 100 U/mL). After being cultured for 24 hours, the cells were treated with quercetin at different concentrations (0, 1, 2, 5 and 10 μM) and cultured for 12 hours, 1, 4 and 7 days, respectively. At each time point, the cells were washed twice with PBS, and then 200 μL DMEM supplemented with 20 μL 5 mg/mL MTT (Amresco, USA) solution was added and incubated at 37°C for 4 hours. Subsequently, the medium was replaced with 200 μL DMSO and vibrated for 10 min to dissolve the MTT formazan crystals. Finally, the absorbance was measured at 490 nm using an ELx Ultra Microplate Reader (BioTek, USA). Meanwhile, a standard curve for the MTT assay was generated; the cells were seeded in 96-well plates at different densities of 5×10^3^, 1×10^4^, 1.5×10^4^, 2×10^4^ and 2.5×10^4^ cells/well and cultured in the DMEM medium, respectively, after which the cell number was quantified according to a cell standard curve [35]. All experiments were performed in triplicate.

### Cell viability analysis

The rBMSCs from passage 1 were seeded in 6-well plates at a density of 1×10^5^ cells/well and cultured in DMEM medium with different concentrations of quercetin at 0, 1, 2, 5 and 10 μM. Cell viability analysis was then performed at 24 hours. The cells were washed twice in PBS and dissociated with 0.25% trypsin/EDTA, after which the cells were collected and stained using an Annexin V-FITC Apoptosis Detection Kit (Beyotime, Shanghai, China) according to the manufacturer’s protocol, and finally analyzed using a fluorescence activated cell sorter (FACS) flow cytometer (Becton–Dickinson, Franklin Lakes, NJ, USA). All experiments were performed in triplicate.

### ALP staining and quantitative analysis

rBMSCs were seeded in 6-well plates at a density of 1×10^5^ cells/well and cultured in DMEM with quercetin at different concentrations, as described above. ALP staining was performed at day 10. Briefly, the cells were treated with BCIP/NBT solution (Beyotime, Shanghai, China) in the dark, and areas stained purple were regarded as positive [36]. Moreover, at days 4, 7 and 10, ALP activity was quantitated following the manufacturer’s instructions (Beyotime, China). Briefly, the cells were washed twice with PBS; 400 μL lysis buffer was added and incubated at 37°C for 4 hours, and the samples were then vibrated for 30 min. Next, each sample was respectively mixed with p-nitrophenyl phosphate disodium (p-NPP) and substrate buffer, vibrated for 10 min and incubated at 37°C for 15 min. The reaction was stopped by the addition of stop buffer to the reaction mixture. ALP activity was quantified by reading the absorbance at 405 nm (BioTek, USA) and calculated according to a standard products. The total protein content in aliquots of the same samples was measured by the Bradford method using the Bio-Rad protein assay kit (Bio-Rad, USA); the absorbance was measured at 630 nm, and protein concentrations were calculated according to a series of BSA (Sigma, USA) standards [37]. ALP activity was normalized to the total cellular protein concentration. All experiments were performed in triplicate.

### Calcium deposition assay

rBMSCs were seeded in 24-well plates at a density of 2.5×10^4^ cells/well and cultured in both basal DMEM medium and osteogenic medium with quercetin at different concentrations (0, 1, 2, 5 and 10 μM), as previously described. Alizarin red-S (ARS) staining was performed at day 28. Briefly, the cells were washed three times in PBS and fixed in 75% ethanol for 30 min. The cells were then stained with ARS solution (40 mM, pH 8.8) for 30 min at 37°C. Moreover, calcium concentration analysis was performed using a calcium colorimetric assay kit (Sigma, USA) according to the manufacturer’s instruction, at days 21 and 28. The calcium concentration was determined using a standard curve and was further normalized to the total cellular protein content, as described above. All experiments were performed in triplicate.

### Real-time quantitative PCR (RT-PCR) analysis

Total cellular RNA was isolated from cells cultured with different concentrations of quercetin for 1, 3, 6, 12 and 24 hours, as previously described. At each time point, the cells were washed twice with PBS, and RNA was extracted using TRIzol reagent (Invitrogen, Carlsbad, CA, USA). The RNA was first separated into an aqueous phase by adding chloroform and was then precipitated with isopropanol. The RNA precipitate was rinsed with 70% ethanol and treated with the RNase inhibitor diethyl pyrocarbonate (DEPC, Sigma) and was finally solubilized in sterile DEPC water. Complementary DNA (cDNA) was then synthesized using a Prime-Script RT reagent kit (Takara Bio, Japan) according to manufacturer’s recommendations. Highly purified gene-specific primers for Runx2, COL1, BSP, BMP-2, OPN, OCN, VEGF, angiogenin-1 (ANG-1) and the housekeeping gene glyceraldehyde-3-phosphate dehydrogenase (GAPDH) were commercially synthesized (Shengong, China). Quantification of the cDNA of bone marker genes was performed with a Bio-Rad My^iQ^ single-color real-time PCR system. All experiments were performed in triplicate.

### Enzyme-linked immunosorbent assay (ELISA)

To analyze the angiogenic protein expression of cells cultured in DMEM with quercetin at 0 μM (control group) and 2 μM (experimental group) concentrations, the VEGF content was measured at days 4, 7 and 10. A VEGF ELISA Kit (Bender, USA) was used to measure the VEGF content according to the manufacturer’s instructions. The VEGF concentration was specifically measured using a standard curve and was further normalized to the total cellular protein content, as described above. All experiments were performed in triplicate.

### Western blotting analysis

For western blotting, rBMSCs were seeded in 6-well plates at a density of 1×10^5^ cells/well and cultured in DMEM with 2 μM quercetin for 0, 30, 60, 90, 120 and 150 min. The cells were then collected and lysed with a protein extraction reagent containing protease inhibitor cocktail, phosphatase inhibitor cocktail and phenylmethanesulfonyl fluoride (PMSF) (Kangchen, China). Equal amounts of protein samples were separated on duplicate 8% SDS-PAGE gels and transferred to polyvinylidene difluoride (PVDF) membranes (Millipore, USA). The membranes were blocked with 5% skim milk and incubated with the appropriate primary antibodies, including rabbit anti-rat ERK, p38, JNK, phosphorylated-ERK (p-ERK), phosphorylated-p38 (p-p38) and phosphorylated-JNK (p-JNK) (dilution, 1:1000; CST, USA), and mouse anti-rat actin (dilution, 1:5000; Sigma, USA). The membranes were then washed three times with PBS containing 0.1% tween-20 detergent and incubated for 2 hours with horseradish peroxidase (HRP)-conjugated goat anti-rabbit and rabbit anti-mouse secondary antibodies (Beyotime, China). Finally, the protein bands were visualized using ECL plus reagent (Amersham Pharmacia Biotech, USA) on a UVItec ALLIANCE 4.7 gel-imaging system. All experiments were performed in triplicate.

### ERK and p38 inhibitor treatment analysis

To identify whether ERK and p38 signaling pathways activation was involved in the observed stimulation of osteogenic differentiation as well as the expression of angiogenic factors by quercetin, quercetin-treated rBMSCs (2 μM) were cultured in the DMEM medium containing either the ERK signaling pathway inhibitor PD98059 (CST, USA) or the p38 signaling pathway inhibitor SB202190 (CST, USA) at a final concentration of 10 μM, respectively. Meanwhile, quercetin-treated rBMSCs cultured without ERK and p38 inhibitors were used as the control group. After being cultured for 150 min, protein was extracted from each group, and western blotting for actin, ERK, p-ERK, p-38 and p-p38 protein levels was performed. Additionally, the gray value of each band was measured using ImageLab software version 4.1 (Bio-Rad). Equal loading of sample controls were used, and the linear range of each protein and the saturation point of the gel for each particular protein was determined. The quantitative assay was then performed according to the linear range [38]. More importantly, the ALP staining at 10 days and real-time PCR analysis for Runx2, Col I, BSP, BMP-2, OPN, OCN, VEGF and ANG-1 expression at 24 hours were respectively performed as previously described. All experiments were performed in triplicate.

### Statistical analysis

All measurements were presented as the mean ± SD. Differences between groups were analyzed by one-way analysis of variance based on the results of the normal distribution and equal variance assumption test [39]. The statistical analyses were conducted using SAS 8.0 software (SAS Inc., USA). A difference was considered statistically significant at a p-value < 0.05 (*^▲^ p < 0.05).

## Results

### MTT and cell viability analysis

**Fig 1:**
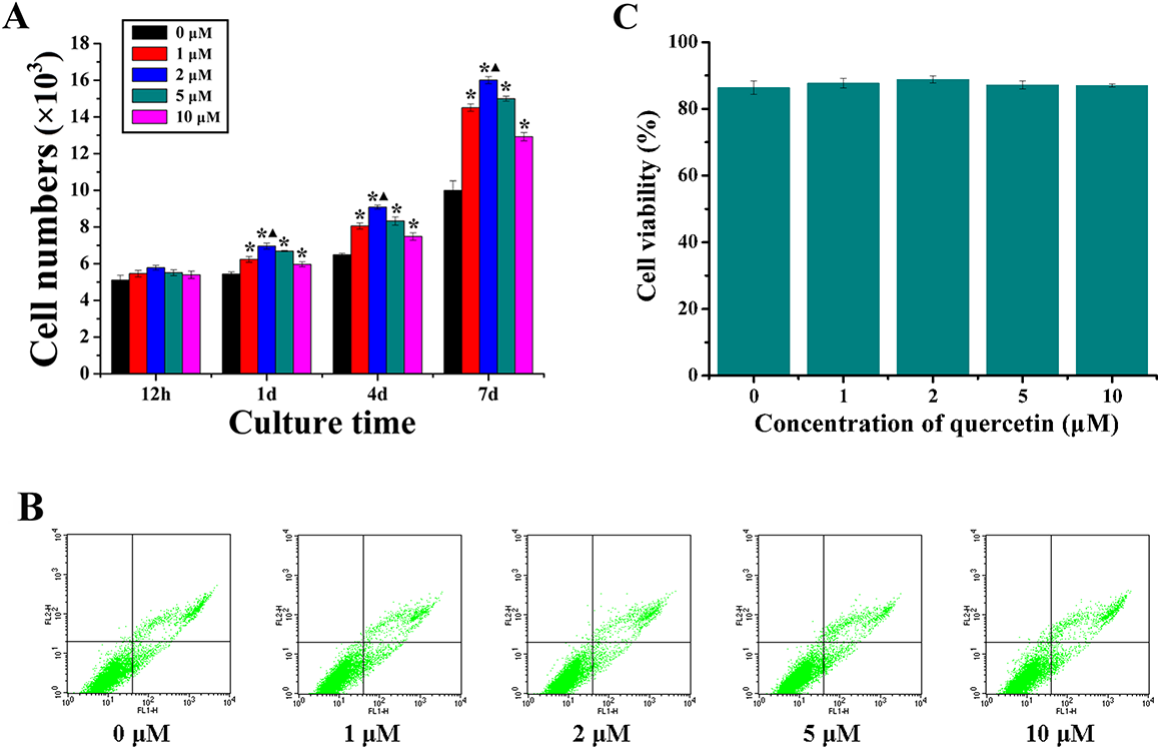
The MTT assay and the cell viability assay. (A) The effect of quercetin at different concentrations (0, 1, 2, 5 and 10 μM) on the proliferation of rBMSCs; (B) The cell viability assay analysed by FACS flow cytometer, the lower-left corner represented as the normal living cells, while the upper-left, lower-right and upper-right corners represented as the detection error, early apoptotic cells, necrotic and late apoptotic cells, respectively; (C) Quantitative analysis of the percentage of normal living cells of rBMSCs cultured with quercetin at different concentrations (0, 1, 2, 5 and 10 μM) at 24 hours, the 0 μM group was treated as the control group (0). *p < 0.05 indicates the quercetin-treated groups vs the control group; ▲p < 0.05 indicates the 2 μM group vs the other quercetin groups.

The MTT assay was performed to compare the proliferation of rBMSCs after being cultured in DMEM medium with different concentrations of quercetin for 12 hours, 1, 4 and 7 days (Fig 1A). Clearly, the cell number increased with increasing culture time, and there were no significant differences between the groups after 12 hours of culture. However, the cell proliferation of the rBMSCs significantly increased in the quercetin-treated groups compared with the respective 0 μM groups at days 1, 4 and 7. Moreover, a significant difference was also observed between the 2 μM group and the other quercetin groups at days 1, 4 and 7 (p < 0.05). Additionally, to explore whether the quercetin treatment induced any cell death, the cell viability analysis was performed after 24 hours. As shown in Fig 1B, the lower-left corner represents the normal living cells, and the upper-left, lower-right and upper-right corners represent detection error, early apoptotic cells, necrotic and late apoptotic cells, respectively. However, no significant differences were observed between the groups (Fig 1C and S2 Fig). Moreover, the cell viability was also measured by trypan blue staining at 24 hours. And the result showed that there were no significant differences observed between the groups (S1 Fig). The cellular morphology was detected at 6 and 24 hours by the actin assay. It can be seen that there were no obvious morphological differences between the groups (S3 Fig).

### ALP activity and calcium deposition assays

**Fig 2:**
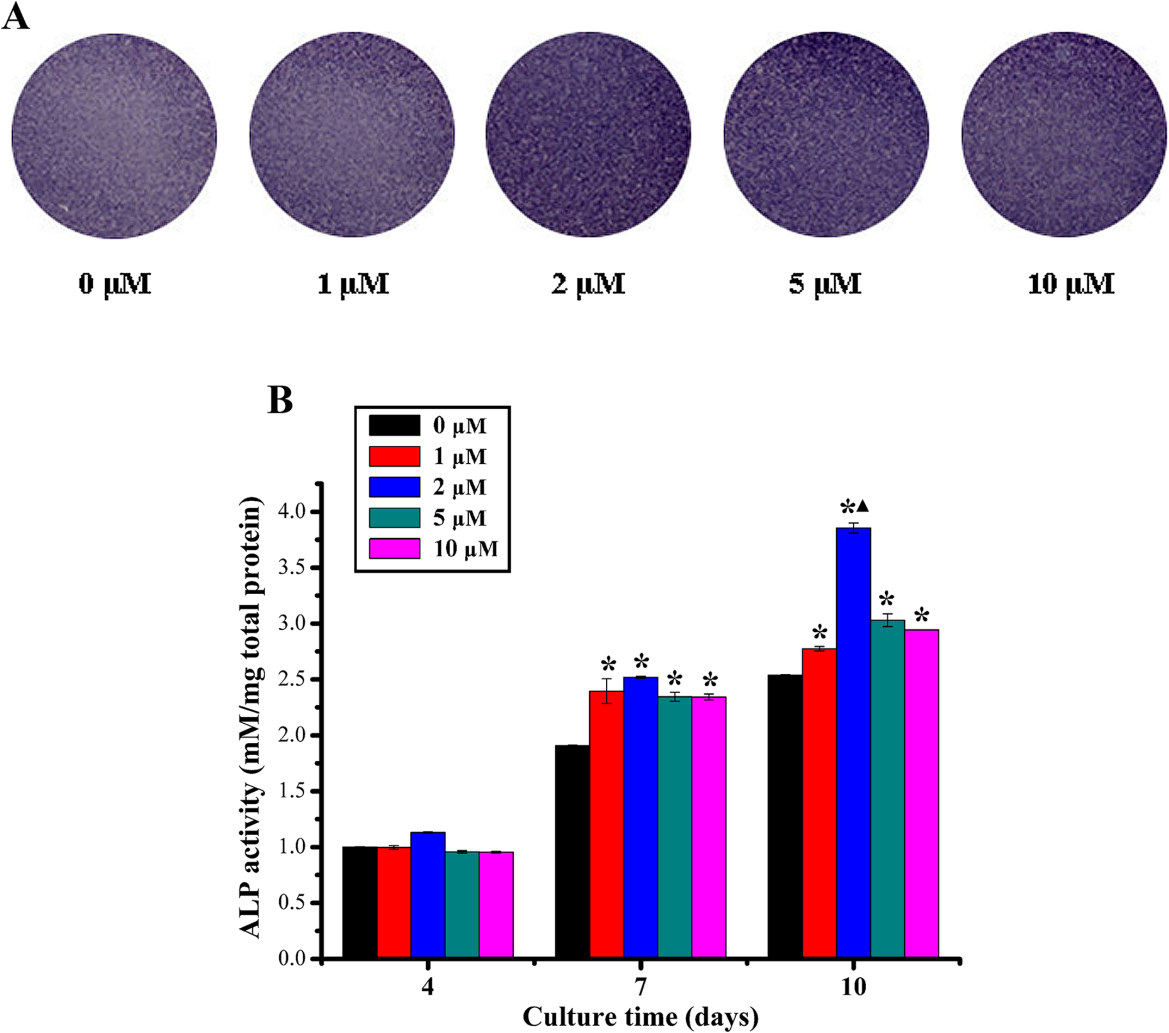
The ALP activity analysis. (A) ALP staining of rBMSCs cultured in DMEM with quercetin at different concentrations (0, 1, 2, 5 and 10 μM) for 10 days; (B) Quantitative analysis of ALP activity of rBMSCs cultured in DMEM with quercetin at different concentrations (0, 1, 2, 5 and 10 μM) for 4, 7 and 10 days. *p < 0.05 indicates quercetin-treated groups vs the control group; ^▲^p < 0.05 indicates the 2 μM group vs the other quercetin groups.

**Fig 3:**
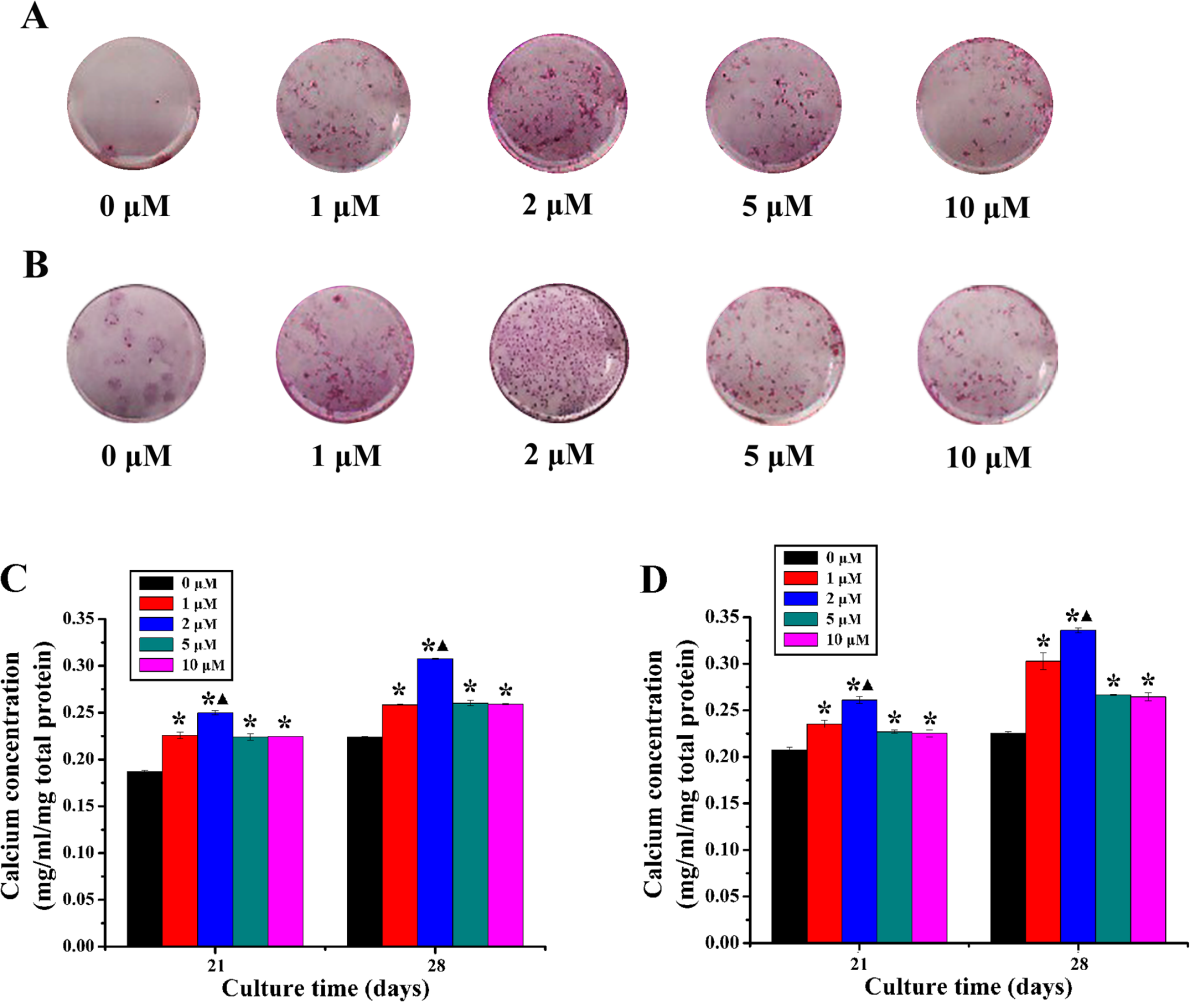
The calcium deposition analysis. (A-B) ARS staining of rBMSCs cultured in DMEM medium (A) and osteogenic medium (B) with quercetin at different concentrations (0, 1, 2, 5 and 10 μM) for 28 days; (C-D) Calcium concentration analysis of rBMSCs cultured in DMEM medium (C) and osteogenic medium (D) with quercetin at different concentrations (0, 1, 2, 5 and 10 μM) for 21 and 28 days. *p < 0.05 indicates the quercetin-treated groups vs the control group; ▲p < 0.05 indicates the 2 μM group vs the other quercetin groups.

The ALP activity of rBMSCs cultured with different concentrations of quercetin was examined. As shown in Fig 2A, ALP staining in the 2 μM, 5 μM and 10 μM quercetin-treated groups were more intense than that in the 0 μM group at day 10. The results of quantitative analysis revealed that the ALP activity in all groups apparently increased over time throughout the assay period and that the ALP activity was highest value at day 10 for the cells cultured with 2 μM quercetin possessed with a statistically significant difference (p < 0.05, Fig 2B). For ARS staining, as shown in Fig 3A and 3B, staining in the quercetin-treated groups, especially in the 2 μM group, was more intense than in the 0 μM group both in DMEM medium and osteogenic medium at day 28. In addition, the results of the calcium concentration analysis showed that the calcium concentration in quercetin-treated groups, especially in the 2 μM group, was significantly higher than that in the 0 μM group both in DMEM medium and osteogenic medium (p < 0.05, Fig 3C and 3D).

### RT-PCR for osteogenic and angiogenic genes

**Fig 4:**
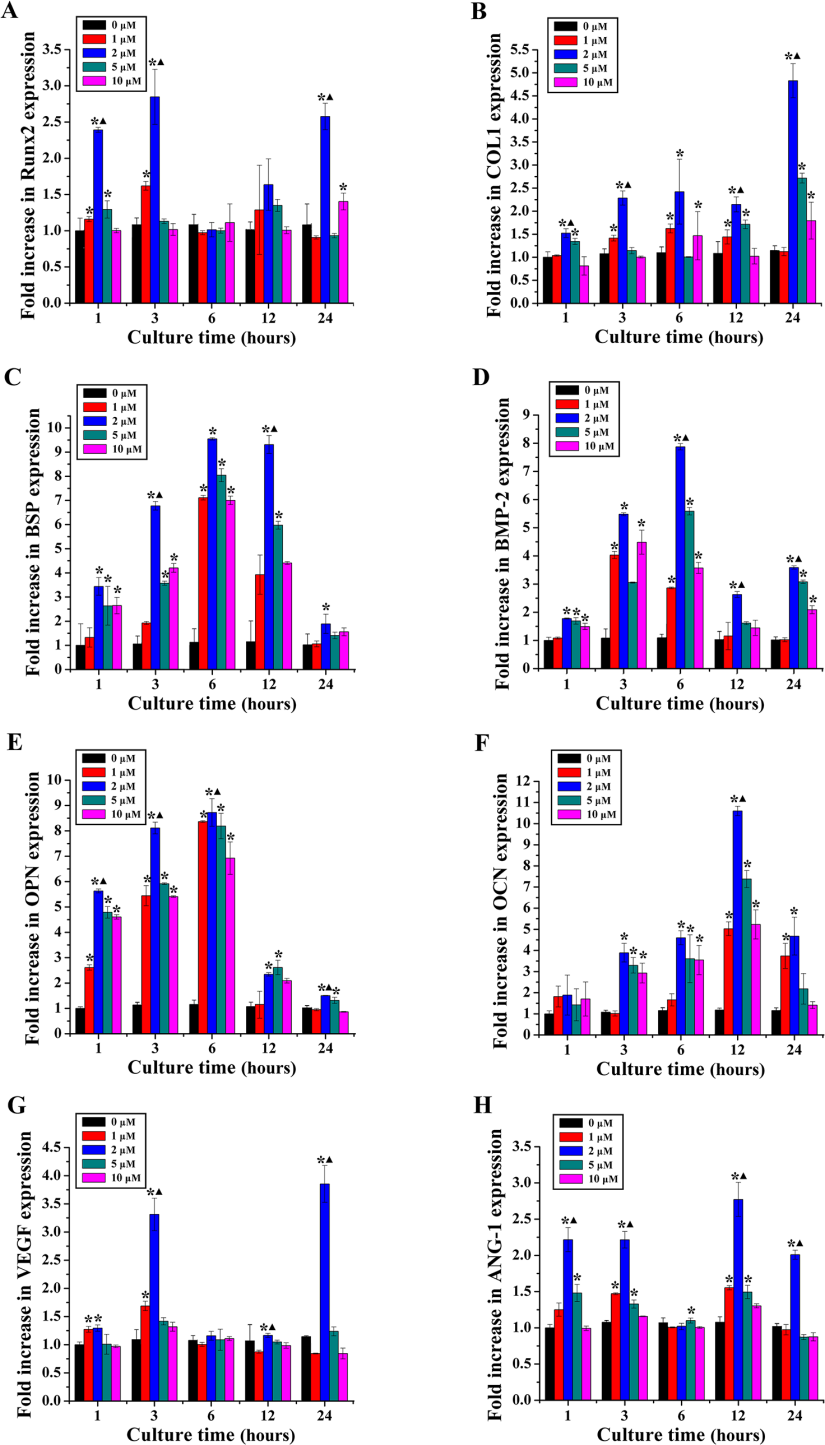
Osteogenic and angiogenic gene expression analysis. The osteogenic and angiogenic genes expression of rBMSCs cultured in DMEM (0 μM) and quercetin-treated groups (1, 2, 5 and 10 μM) for 1, 3, 6, 12, 24 hours. (A) Runx2; (B) COL1; (C) BSP; (D) BMP-2; (E) OPN; (F) OCN; (G) VEGF; (H) ANG-1. *p < 0.05 indicates the quercetin-treated groups vs the control group; ^▲^p < 0.05 indicates the 2 μM group vs the other quercetin groups.

Runx2, COL1, BSP, BMP-2, OPN, OCN, VEGF and ANG-1 gene expression was examined by RT-PCR after rBMSCs were cultured in DMEM with different concentrations of quercetin (0, 1, 2, 5 and 10 μM) for 1, 3, 6, 12 and 24 hours (Fig 4). The expression of Runx2 in the quercetin-treated groups peaked at 3 hours; the increasing trend slowed down at 6 and 12 hours. Additionally, the expression of COL1 in the quercetin-treated groups was higher than that in the 0 μM group at each time point and was highest at 24 hours. Meanwhile, the expression of BSP in the quercetin-treated groups was significantly increased before 24 hours compared with that in the 0 μM group. The expression of BMP-2 in the quercetin-treated groups was higher than that in the 0 μM group at 3, 6 and 24 hours, whereas the expression of OPN in the quercetin-treated groups was remarkably higher than that in the 0 μM group at 1, 3 and 6 hours and peaked at 6 hours. Moreover, the expression of OCN in the quercetin-treated groups was significantly increased after 1 hour compared with that in the 0 μM group and peaked at 12 hours. Interestingly, the expression of VEGF in the quercetin-treated groups was remarkably increased at 3 and 24 hours compared with that in the 0 μM group. Moreover, the expression of ANG-1 in the quercetin-treated groups was significantly higher than that in the 0 μM group at 1, 3, 12 and 24 hours. Importantly, this stimulatory effect was achieved in a dose-dependent manner, whereas the concentration of 2 μM achieved the highest stimulatory effect. Moreover, a PT-PCR analysis was performed at 1 week and the results showed that 2 μM quercetin group can significantly stimulate the expression of Runx2, BMP-2, OPN, OCN and VEGF than the other groups (p < 0.05, S4 Fig). Therefore, the concentration of 2 μM was chosen as the optimum drug concentration for the following studies.

### VEGF protein content

**Fig 5:**
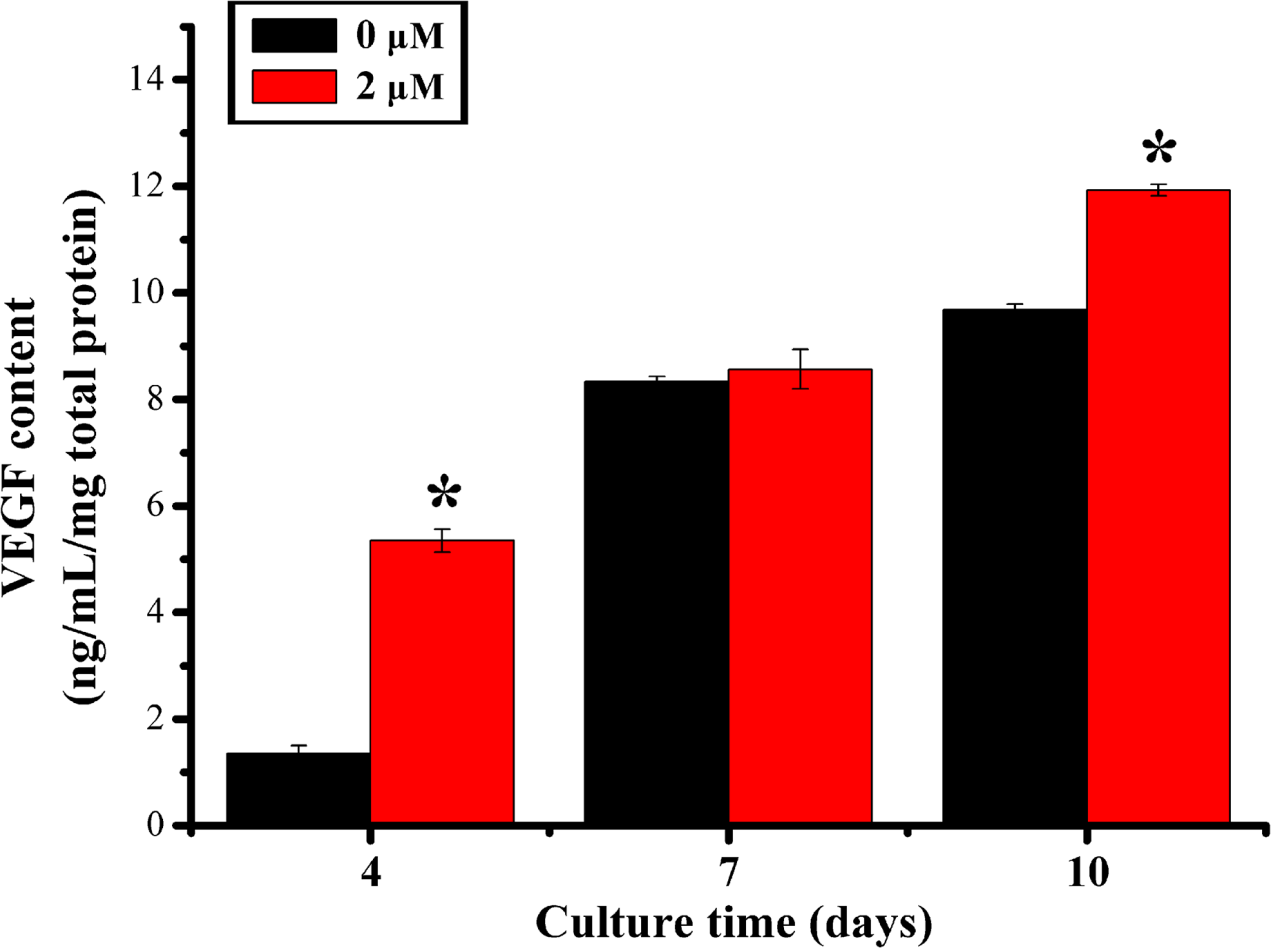
VEGF protein content test by ELISA assay. The protein level of VEGF in the DMEM medium group (0 μM, control group) and quercetin-treated group with the optimum concentration (2 μM) at days 4, 7 and 10. *p < 0.05 indicates the 2 μM quercetin-treated group vs the control group.

The amount of VEGF protein released from rBMSCs cultured in DMEM with 0 μM and 2 μM quercetin was measured by ELISA on days 4, 7 and 10. The results showed that the quercetin significantly increased the VEGF protein level on days 4 and 10 (p < 0.05, Fig 5).

### Western blotting analysis of MAPK signaling pathways

**Fig 6:**
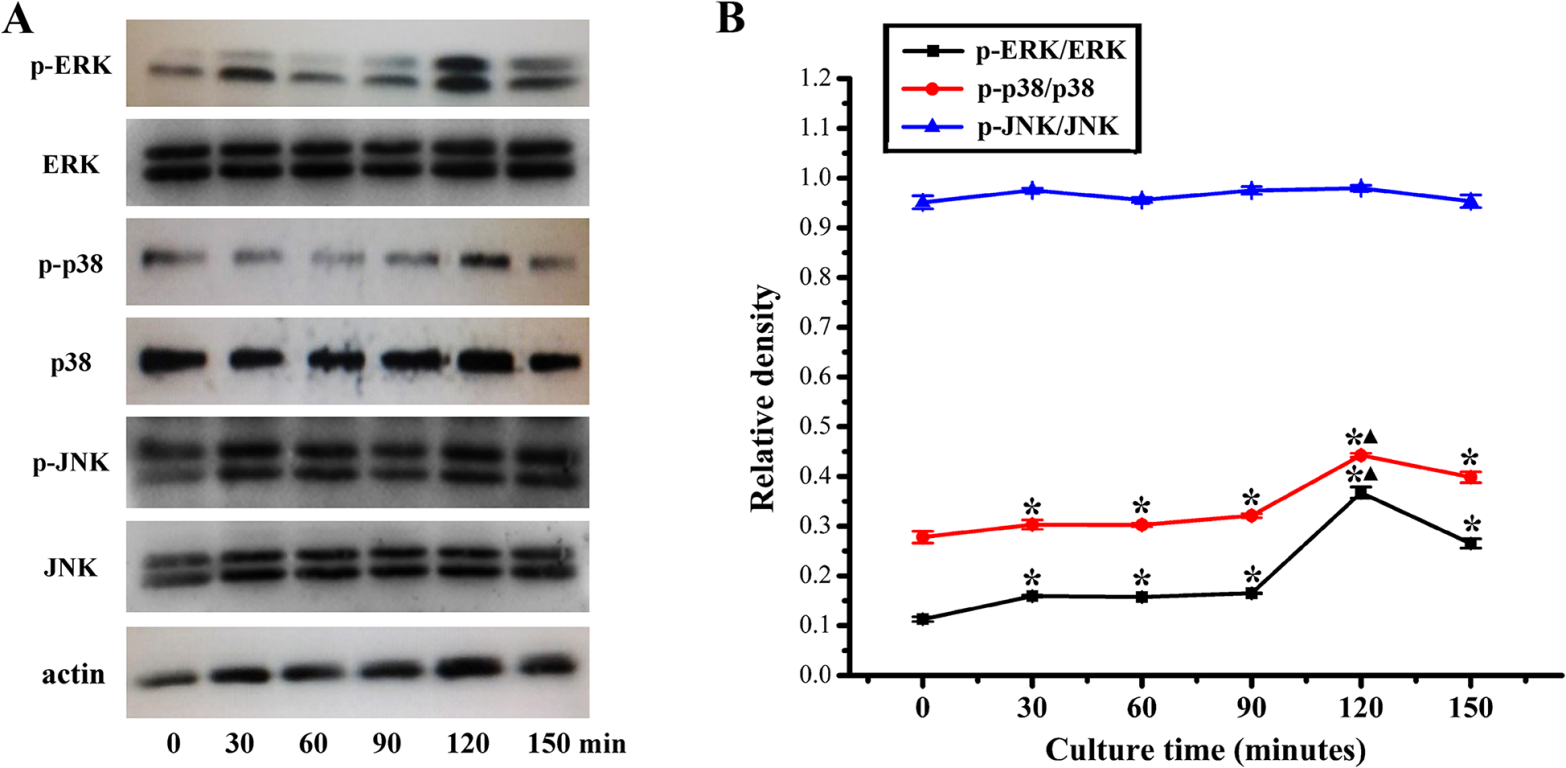
Analysis of phosphorylation level of ERK, p38 and JNK in quercetin-treated rBMSCs by western blotting. (A) Western blotting for the protein levels of ERK, p38 and JNK signaling pathways in the cells cultured with 2 μM quercetin for 0, 30, 60, 90, 120 and 150 min; (B) The ratios of p-ERK/ERK, p-p38/p38 and p-JNK/JNK were calculated based on the relative band densities, respectively. *p < 0.05 indicates the quercetin-treated groups vs the 0 min group; ^▲^p < 0.05 indicates the 120 min group vs the other groups.

Western blotting analysis was performed to measure the protein levels of p-ERK, p-p38, p-JNK, ERK, p38, JNK and actin in the total protein extracted from rBMSCs treated with 2 μM quercetin for 0, 30, 60, 90, 120 and 150 min. As shown in Fig 6, quercetin could activate the ERK and p38 signaling pathways, and remarkable phosphorylation of ERK and p38 was observed at 120 min; however, no significant activation of the JNK signaling pathway was observed.

### Inhibition of the ERK and p38 signaling pathways

**Fig 7:**
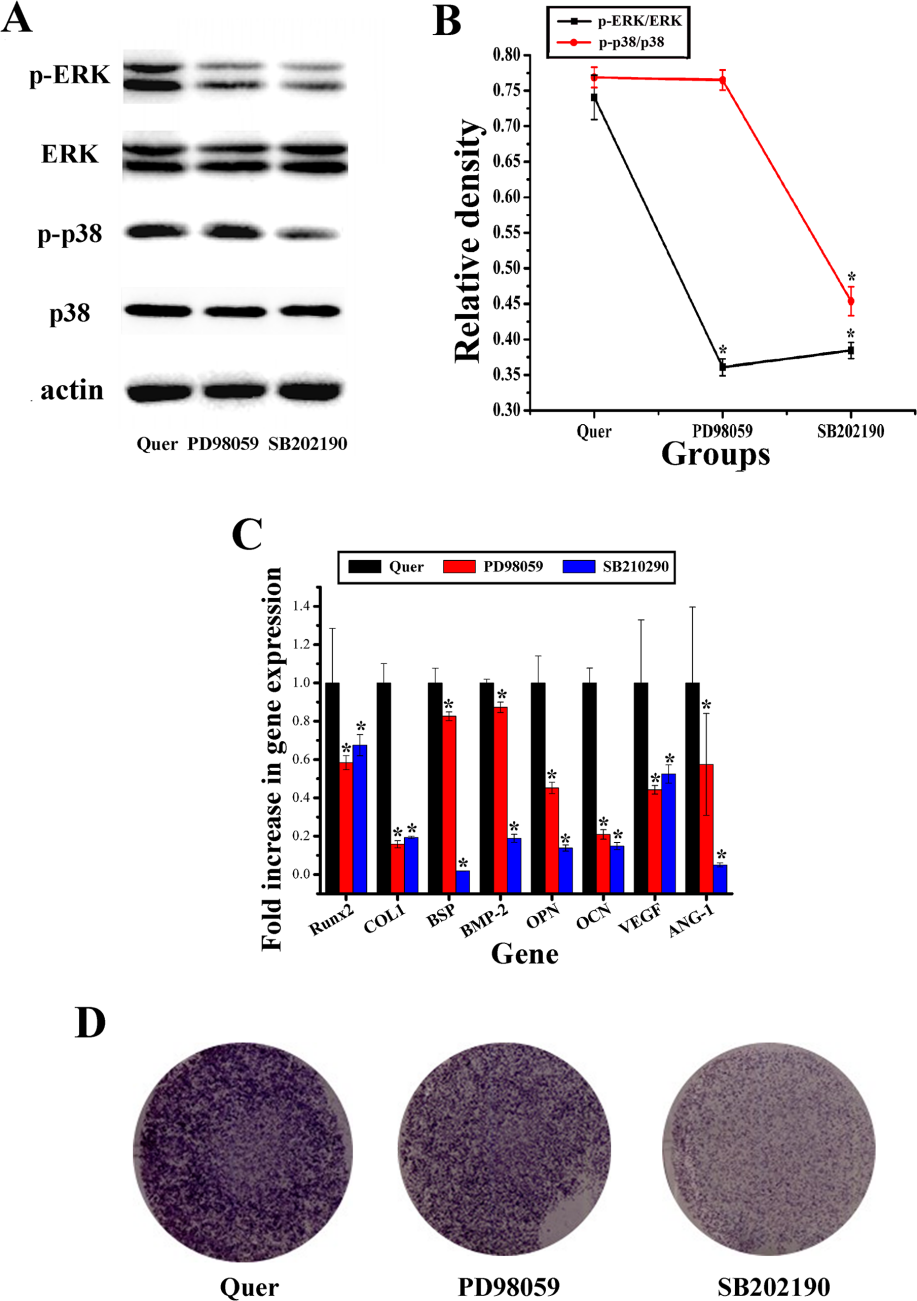
Signaling pathways inhibition analysis. (A) Western blotting for the protein levels of the ERK and p38 signaling pathways in cells in the 2 μM quercetin-treated group (Quer), the inhibitor PD98059 group and inhibitor SB202190 group at 150 min; (B) The ratios of p-ERK/ERK and p-p38/p38 were calculated based on the relative linear ranges, respectively; (C) RT-qPCR analysis of osteogenic and angiogenic genes at 24 hours after the rBMSCs were cultured with 2 μM quercetin and the ERK signaling pathway inhibitor PD98059 (10 μM), or the p38 signaling pathway inhibitor SB202190 (10 μM), respectively; (D) ALP staining of quercetin-treated rBMSCs cultured with the ERK signaling pathway inhibitor PD98059 (10 μM) or the p38 signaling pathway inhibitor SB202190 (10 μM) at day 10. The cells cultured in 2 μM quercetin without inhibitors were set as the control group, which is labeled as “Quer”. *p < 0.05 indicates the inhibitor PD98059 or the inhibitor SB202190 group vs the quercetin group.

To assess the roles of the ERK and p38 signaling pathways in the process of the stimulatory effect of quercetin, the ERK signaling pathway inhibitor PD98059 (10 μM) and the p38 signaling pathway inhibitor SB202190 (10 μM) were used on the cells treated with quercetin. The result showed that phosphorylation of ERK and p38 was depressed by the inhibitors of PD98059 and SB202190, correspondingly; moreover, the inhibitor SB202190 could also inhibit phosphorylation of ERK signaling pathway in the present study (Fig 7A and 7B). Moreover, Runx2, COL1, BSP, BMP-2, OPN, OCN, VEGF and ANG-1 gene expression in quercetin-treated cells cultured with PD98059 or SB202190 was also significantly repressed compared with that of cells treated with quercetin alone (Fig 7C). Furthermore, ALP staining was also obviously weakened under treatment with PD98059 and SB202190 (Fig 7D). Analysis of these results demonstrated that the ERK and p38 signaling pathways played an important role in the process of enhanced effect of quercetin on rBMSCs.

## Discussion

BMSCs have been widely used for bone regeneration for a long time [36]. Sources of BMSCs are widespread, and these cells are easy to obtain and can undergo multipotential differentiation [40]. Thus, BMSCs are one of the most commonly used types of seed cells for bone tissue engineering. Under certain conditions, BMSCs can differentiate into osteogenic cells and stably express an osteoblastic phenotype; moreover, their osteogenic activities can continue after implantation [41]. Due to the limited availability of human MSC tissue sources, rat MSCs were chosen in the present study; however, the use of these cells limits the clinical relevance of the results, which means the findings should be validated by using human primary MSCs in our future work on quercetin.

Interestingly, using a specific certain induction method, BMSCs could secrete angiogenic factors, which facilitate the function of angiogenesis. It is well known that angiogenesis is important for subsequent bone regeneration in vivo. Previous studies have shown that the survival of seed cells in vivo relies on the blood supplying of oxygen and nutrients and that a lack of oxygen and nutrition can cause the rapid necrosis or apoptosis of cells within 3 days [42]. Therefore, the present study aimed to identify an ideal induction method to promote the osteogenic differentiation of BMSCs and the secretion of angiogenic factors by these cells.

As a traditional Chinese medicine, quercetin is commonly used to treat scurvy and heart disease [24, 25]. Moreover, recent studies have shown that quercetin can promote osteogenic differentiation [29]. According to previous studies, quercetin could obviously promote the osteogenic differentiation of human ASCs at a concentration of 2 μM, whereas a concentration of 5 μM was optimal for osteoblast-like cells [26, 27]. These findings indicate that the optimal quercetin concentration may be cell type dependent. Therefore, based on previous studies, the concentrations of 1, 2, 5 and 10 μM were selected in the present study. Analysis of the in vitro results suggested that quercetin could enhance the osteogenic differentiation of rBMSCs as well as the expression of angiogenic factors in a dose-dependent manner and that a concentration of 2 μM could achieve the greatest stimulatory effect.

Previous studies showed that quercetin could stimulate the osteogenic differentiation of ASCs and osteoblastic cells but that there was no positive effect on cell proliferation [26–28]. In the present study, the effect of quercetin on the proliferation and osteogenic differentiation of BMSCs was comprehensively studied. In contrast to previous studies, our results indicated that quercetin could enhance the proliferation of rBMSCs at days 1, 4 and 7 in a dose-dependent manner, whereas a concentration of 2 μM could achieve the greatest effect; furthermore, quercetin did not affect cell viability or cellular morphology. Following quercetin treatment, ALP activity and calcium deposition assays and osteogenic gene expression analysis were performed. ALP, an early marker of osteogenic differentiation, is positively correlated with cell differentiation and maturation [43, 44], whereas calcium deposition is a later marker of osteogenic differentiation [45]. It is well known that Runx2 and COL1 are considered as early markers of osteogenic differentiation [46], whereas BSP, BMP-2 and OCN are middle and late markers [47–49]. OPN appears during early proliferation and later differentiation [50, 51]. It has been reported that Runx2 is involved in the regulation of gene expression during the process of osteogenic differentiation, which plays an important role in the maturation and stabilization of osteoblasts [52]. COL1 provides a basic framework for mineralized inorganic material deposition and plays a decisive role in the biomechanical strength properties of bone tissue [53]. BSP plays a key role in the initiation processes of bone matrix mineralization and mineralized cell adhesion matrix formation [54]. Moreover, BMP-2 can promote the proliferation and osteogenic differentiation of BMSCs and can also inhibit the differentiation of BMSCs into other cell types, such as adipose and skeletal muscle cells [55, 56]. As a bone matrix component, OCN is one of the main indicators of the phase of osteoblast differentiation involving mineralization, whereas OPN is related to the phase of osteoblasts maturation between adhesion prior to mineralization and matrix synthesis [57, 58]. In the present study, as an early marker of osteogenic differentiation, the expression of Runx2 in the 2 μM group increased at 1 hour and peaked at 3 hours; this increase in expression of Runx2 then slowed down at 6 and 12 hours. In accordance with reports indicating that Runx2 can stimulate OPN, OCN and BSP gene expression [59], OPN, OCN and BSP gene expression in the 2 μM group peaked at 6 and 12 hours, respectively, in the present study. Moreover, it has also been suggested that the enhanced gene expression of these osteogenic genes could consequently increase Runx2 gene expression [60]. In the present study, Runx2 gene expression in the 2 μM group was significantly different from that in the other groups at 24 hours, possibly due to the stimulatory effects of OPN, OCN and BSP. However, in the present study, quercetin could enhance ALP activity and the above osteogenic genes, especially the middle and late markers (BSP, BMP-2, OPN and OCN), in a dose-dependent manner; this stimulatory effect was most obvious at the concentration of 2 μM. These results indicated that quercetin could not only promote the proliferation of rBMSCs but also enhance osteogenic differentiation at various stages, especially at the middle and late stages; additionally, the concentration of 2 μM was found to be the optimum concentration. However, previous studies have shown that the cellular morphology is one of aspects, which can influence the osteogenic differentiation of MSCs [61–63]; the other studies also showed that some transcription factors (TFs) and Chinese medicine, such as zinc fingers and homeoboxes 3 (ZHX3), Runx2 and Tinospora cordifolia, could promote the osteogenic differentiation of MSCs by regulating the gene expression and secretion of osteogenic markers without significant effect on the cellular morphology [64–66]. Present study also showed that quercetin could stimulate the osteogenic differentiation of rBMSCs by promoting the gene expression and secretion of osteogenic markers without obvious effect on the cellular morphology.

Most previous studies regarding quercetin have focused on osteogenic differentiation; however, the effect of quercetin on the expression of angiogenic factors was not evaluated [26, 27]. The present study also analyzed the effect of quercetin on the expression of angiogenic factors by BMSCs. As a key angiogenic factor, VEGF can simultaneously promote osteogenesis and angiogenesis [67, 68], and can specifically affect vascular endothelial cells by inducing cell proliferation, migration and angiogenesis [69, 70]. As a paracrine angiogenic factor, VEGF could also increase the permeability of small veins and venules and promote the accumulation of cytoplasmic calcium, as well as induce angiogenesis [71]. A previous study also showed that VEGF could stimulate the proliferation and osteogenesis of BMSCs and indirectly promote the proliferation and differentiation of osteoblasts by stimulating endothelial cells to secrete osteoanabolic growth factors [72–74]. Acting as another important angiogenic factor, ANG-1 can stimulate angiogenesis by reducing the VEGF-mediated vascular permeability to a certain extent [75]. Additionally, ANG-1 can promote the differentiation, bone matrix deposition and mineralization of osteoblasts [76]. Although previous studies showed that quercetin could inhibit the angiogenic gene expression of tumor cells [77, 78], in the present study, the results of RT-PCR and ELISA analyses showed higher VEGF gene and protein levels in rBMSCs treated with quercetin. Moreover, the results of RT-PCR showed that quercetin could stimulate the gene expression of ANG-1. All of these data indicate that quercetin could enhance angiogenic factor expression of rBMSCs, which could in turn promote angiogenesis and osteogenesis in vivo.

However, previous research has mostly focused on the secretion of angiogenic factors by undifferentiated bone marrow derived MSCs such as mesenchymal progenitor cells, marrow-derived stromal cells and multipotent stromal cells [79–81]; moreover, recent studies reported that the osteogenically differentiated MSCs that cultured in osteogenic induction medium could lead cells antiangiogenic and reduce the secretion of angiogenic factors like VEGF and platelet-derived growth factor-AA (PDGF-AA) [82, 83]. However, many studies reported that some growth factors such as BMP-2 [84, 85], BMP-9 [86], epidermal growth factor (EGF) [87], growth and differentiation factor-5 (GDF-5) [88] and basic fibroblast growth factor (bFGF) [84, 89, 90], could promote osteogenic differentiation of MSCs as well as secretion of angiogenic factors by inducing both osteogenic and angiogenic signaling pathways without osteogenic induction medium. In the present study, it has been firstly observed that as a kind of Chinese medicine, quercetin could not only promote osteogenic differentiation of rBMSCs, but also enhance the secretion of angiogenic factors in a dose-dependent manner, while the concentration of 2 μM could achieve the greatest effect. Recent studies also reported that quercetin could stimulate the induction and functions of immune cells and enhance the functions of the immune system [91, 92]. However, whether quercetin can affect the immunogenic status of BMSCs has remained largely unknown. Indeed, the immunogenicity of MSCs is the one of the important topics in bone regeneration and will be assessed in our future studies.

Previous studies showed that quercetin could promote the osteogenic differentiation of MG-63 human osteoblasts through the ERK signaling pathway [26, 33]; however, it has remained largely unknown whether the p38 and JNK signaling pathways, the other two important pathways in MAPK signaling, are involved in this process. It is well known that the ERK, p38 and JNK pathways are crucial for the regulation of cell proliferation, osteoblast differentiation and skeletal development [93, 94]. In particular, the ERK signaling pathway can be activated in cells throughout the developmental phase, which can promote immediate early gene expression, reinforce matrix mineralization and promote osteogenic differentiation [95]. The p38 signaling pathway plays an important role in cell growth, survival and differentiation [96]. The JNK signaling pathway has been reported to potentially enhance OCN mRNA level and to be involved in BMP-2-induced osteoblastic cell differentiation [97, 98]. Our present findings showed that quercetin could activate the ERK and p38 signaling pathways, resulting in higher p-ERK and p-p38 protein levels at 120 min. However, quercetin did not lead to significant activation of the JNK signaling pathway. Furthermore, to demonstrate whether the stimulatory effect of quercetin on the proliferation and differentiation of rBMSCs is dependent on the ERK and p38 signaling pathways, the specific ERK and p38 inhibitors PD98059 and SB202190 were applied to quercetin-treated rBMSCs, respectively. The results showed that pretreatment with PD98059 and SB202190 blocked quercetin-induced ERK and p38 phosphorylation, respectively. Moreover, quercetin-stimulated ALP activity and the expression of osteogenic genes (Runx2, COL1, BSP, BMP-2, OPN and OCN) and angiogenic genes (VEGF and ANG-1) could be repressed by either PD98059, or SB202190, respectively. Moreover, the inhibitor SB202190 could also inhibit phosphorylation of the ERK signaling pathway in the present study. Previous study also reported that the p38 inhibitor was found to abrogate TNF-alpha-induced ERK phosphorylation in human proximal tubular epithelial cells [99]. It has been suggested that there are several points shared between the ERK and the p38 signaling pathways, leading to cross-talk between these two pathways [100, 101]. Therefore, p38 may also regulate ERK phosphorylation in the context of the effects of quercetin, although this concept requires further evaluation in our future studies. All of these data indicated that quercetin stimulated the proliferation, osteogenic differentiation and angiogenic factor expression of rBMSCs, partially through the ERK and p38 signaling pathways.

In conclusion, quercetin could promote the cell proliferation, osteogenic differentiation and angiogenic factor expression of rBMSCs in a dose-dependent manner, and a concentration of 2 μM quercetin achieved the greatest stimulatory effect. Furthermore, the ERK and the p38 signaling pathways might play an important role in this process. It is suggested that quercetin may represent a drug that can be potentially applied for bone regeneration.

## Supporting Information

S1 Fig Typan blue staining for cell viability. The effect of quercetin at different concentrations (0, 1, 2, 5 and 10 μM) on the cell viability of rBMSCs at 24 hours. The 0 μM group was treated as the control group (0).
(TIF)

S2 Fig FACS flow cytometer for cell apoptosis. Quantitative analysis of the percentages of early apoptotic cells (A) and necrotic/late apoptotic cells (B) of rBMSCs cultured with quercetin at different concentrations (0, 1, 2, 5 and 10 μM) at 24 hours. The 0 μM group was treated as the control group (0).
(TIF)

S3 Fig Cellular morphology. The cellular morphology detected by actin cytoskeletal staining showing at 6 and 24 hours.
(TIF)

S4 Fig Gene expression of rBMSCs treated with different concentrations of quercetin at day 7. The osteogenic and angiogenic genes expression of BMSCs cultured in DMEM medium with quercetin at different concentrations (0, 1, 2, 5 and 10 μM) for 7 days. *p < 0.05 indicates the quercetin-treated groups vs the control group (0 μM); ▲p < 0.05 indicates the 2 μM group vs the other quercetin groups.
(TIF)

S1 Table Primer sequences for real-time quantitative RT-PCR. (DOC)
