# Supplementary figures and images for: The Effect of Quercetin on the Osteogenesic Differentiation and Angiogenic Factor Expression of Bone Marrow-Derived Mesenchymal Stem Cells
Source: PLoS One. 2015 Jun 8;10(6):e0129605. doi: 10.1371/journal.pone.0129605 (PMC4460026; doi:10.1371/journal.pone.0129605)

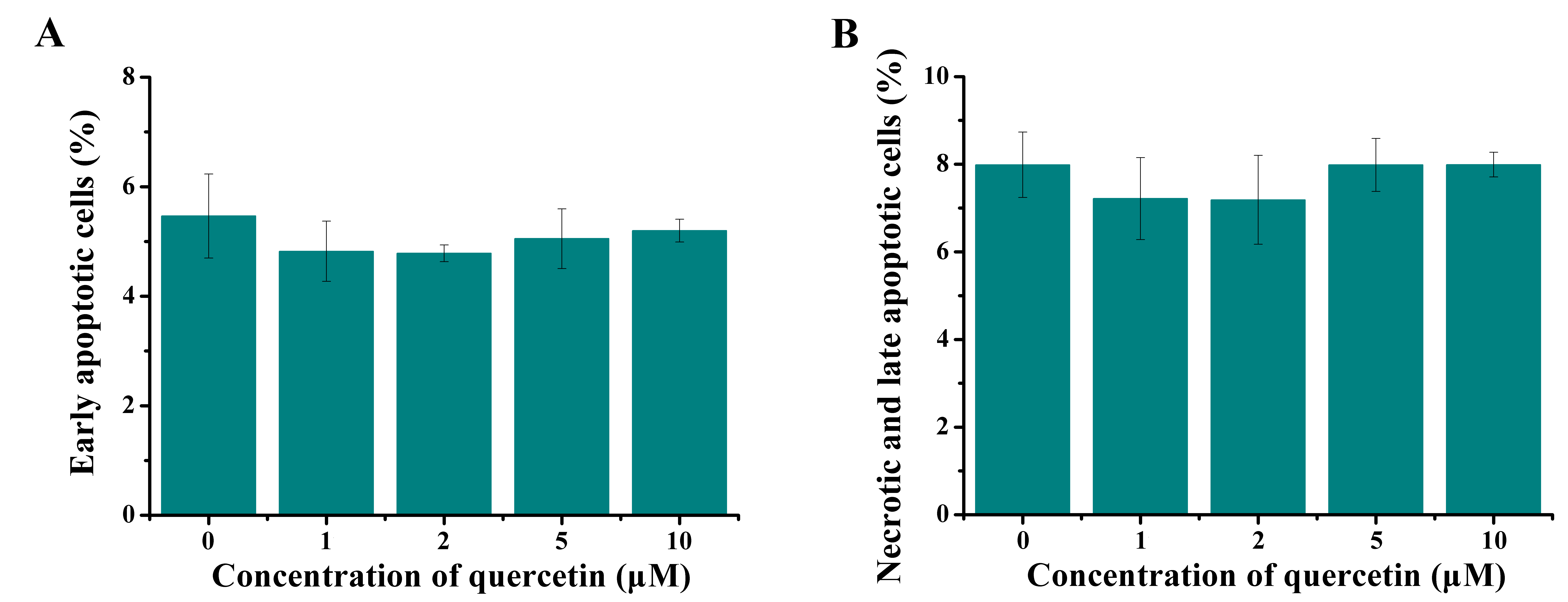

Supplement: S2 Fig — Quantitative analysis of the percentages of early apoptotic cells (A) and necrotic/late apoptotic cells (B) of rBMSCs cultured with quercetin at different concentrations (0, 1, 2, 5 and 10 μM) at 24 hours. The 0 μM group was treated as the control group (0). (TIF) [file pone.0129605.s002.tif]

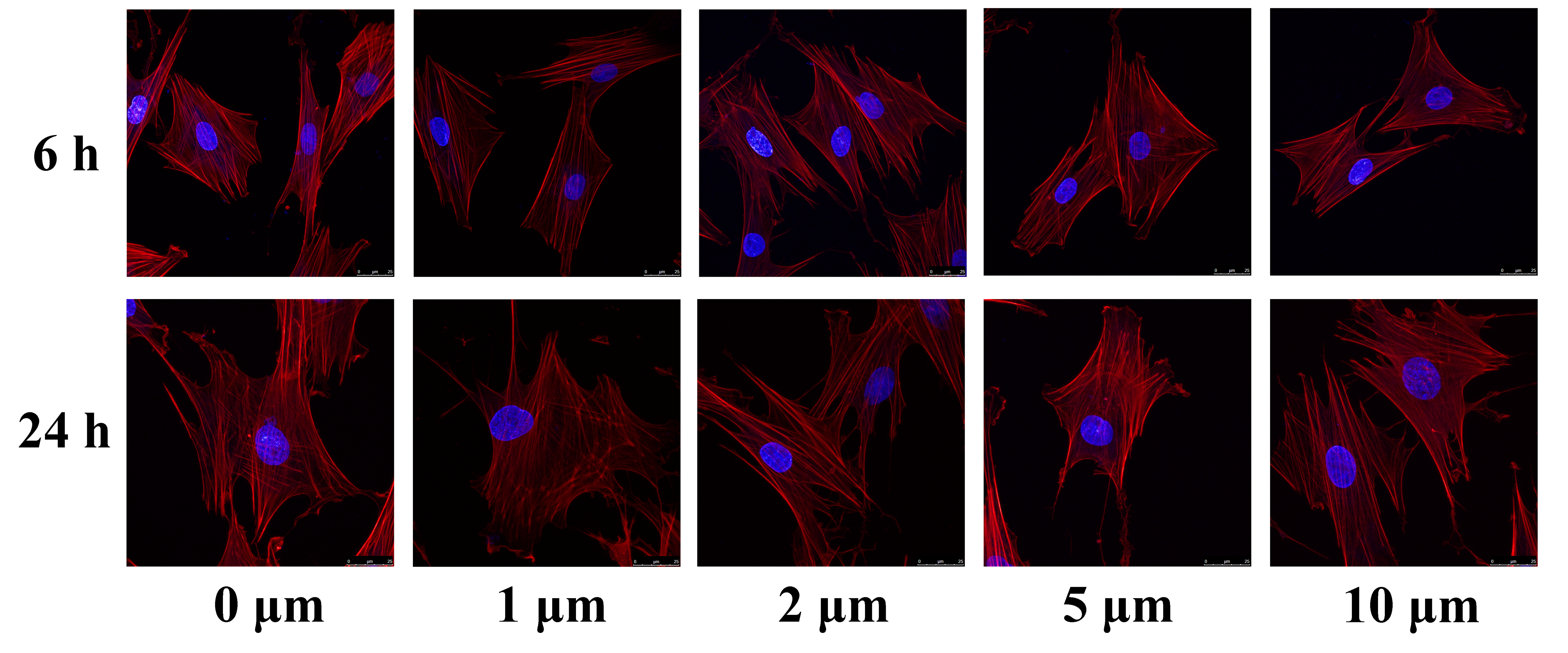

Supplement: S3 Fig — The cellular morphology detected by actin cytoskeletal staining showing at 6 and 24 hours. (TIF) [file pone.0129605.s003.tif]

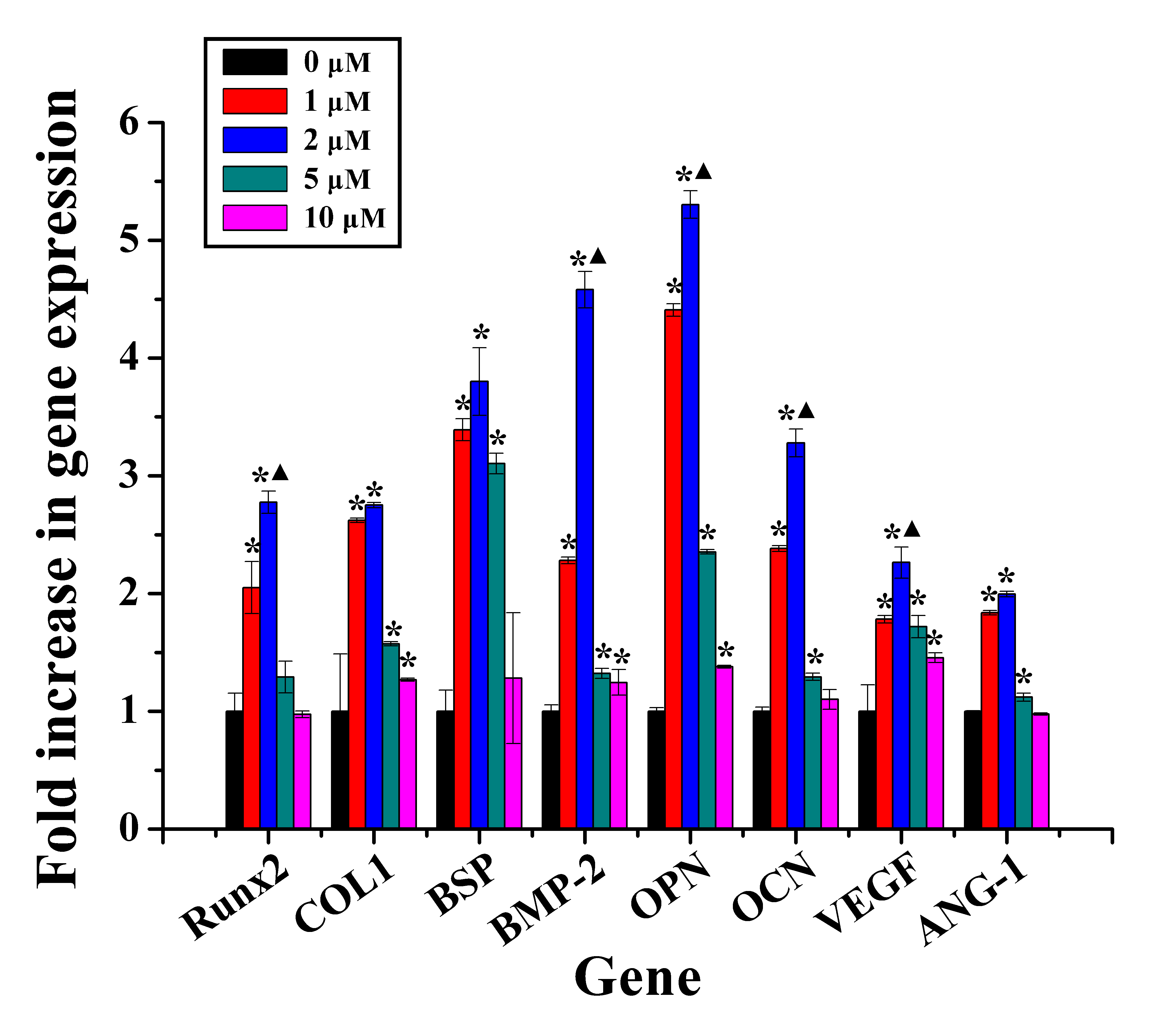

Supplement: S4 Fig — The osteogenic and angiogenic genes expression of BMSCs cultured in DMEM medium with quercetin at different concentrations (0, 1, 2, 5 and 10 μM) for 7 days. *p < 0.05 indicates the quercetin-treated groups vs the control group (0 μM); ▲p < 0.05 indicates the 2 μM group vs the other quercetin groups. (TIF) [file pone.0129605.s004.tif]
