# Supplementary material for: The Effect of Quercetin on the Osteogenesic Differentiation and Angiogenic Factor Expression of Bone Marrow-Derived Mesenchymal Stem Cells
Source: PLoS One. 2015 Jun 8;10(6):e0129605. doi: 10.1371/journal.pone.0129605 (PMC4460026; doi:10.1371/journal.pone.0129605)
Supplement: S1 Table — (DOC) [file pone.0129605.s005.doc]

**Table S1. The primer sequences for real-time quantitative RT-PCR.**

| Gene | Forward (5'-3') | Reverse (5'-3') |
| --- | --- | --- |
| Runx2 | ATCCAGCCACCTTCACTTACACC | GGGACCATTGGGAACTGATAGG |
| COL1 | CAGGCTGGTGTGATGGGATT | CCAAGGTCTCCAGGAACACC |
| BSP | AGAAAGAGCAGCACGGTTGAGT | GACCCTCGTAGCCTTCATAGCC |
| BMP-2 | TGGGTTTGTGGTGGAAGTGGC | TGGATGTCCTTTACCGTCGTG |
| OPN | CCAAGCGTGGAAACACACAGCC | GGCTTTGGAACTCGCCTGACTG |
| OCN | GCCCTGACTGCATTCTGCCTCT | TCACCACCTTACTGCCCTCCTG |
| VEGF | GGCTCTGAAACCATGAACTTTCT | GCAATAGCTGCGCTGGTAGAC |
| ANG-1 | GGACAGCAGGCAAACAGAGCAGC | CCACAGGCATCAAACCACCAACC |
| GAPDH | TTCGACAGTCAGCCGVATCTT | ATCCGTTGACTCCGACCTTCA |
